# Synthetic bioreducible lipid‐based nanoparticles for miRNA delivery to mesenchymal stem cells to induce neuronal differentiation

**DOI:** 10.1002/btm2.10021

**Published:** 2016-08-08

**Authors:** Yuji S. Takeda, Ming Wang, Pu Deng, Qiaobing Xu

**Affiliations:** ^1^ Dept. of Biomedical Engineering Tufts University 4 Colby Street Medford MA 02155; ^2^ Present address: Beijing National Laboratory for Molecular Sciences, Key Laboratory of Analytical Chemistry for Living Biosystems Institute of Chemistry Beijing 100190 China, The Chinese Academy of Sciences (CAS)

**Keywords:** bone marrow stromal cells, combinatorial library, microRNA, neuronal differentiation, synthetic lipid nanoparticles

## Abstract

MicroRNA (miRNA) functions in tissue regeneration and determines the fate of stem cells. Nanoparticle‐based miRNA delivery systems for therapeutic applications have been studied in clinical settings. However, gene delivery to stem cells is still a challenging issue. Lipid‐like nanoparticles produced using combinatorial approaches have recently been used for delivery of a variety of biologics. In this study, we investigated the ability of these lipids to deliver miRNA to human mesenchymal stem cells (hMSCs). First, small library screening of bioreducible lipids was performed using fluorophore‐conjugated miRNA to determine the optimal chemical structure for miRNA delivery to hMSCs. Next, miRNA‐9 (miR‐9), which promotes neuronal differentiation of stem cells, was delivered to hMSCs using the lipids identified from the library screening. Morphological changes of the cells and upregulation of neuronal marker genes were observed after the delivery of miR‐9. The synthetic bioreducible lipids are effective in facilitating miRNA delivery to hMSCs and promoting the neuronal differentiation.

## 1 Introduction

MicroRNA (miRNA) modulates gene expression, functions in tissue regeneration, and determines the fate of stem cells.^1^, ^2^ Several miRNAs, such as miR‐9 and miR‐124, have been identified to play a role in neuronal differentiation of stem cells.^1^, ^3^, ^4^, ^5^, ^6^ Jing et al reported that mesenchymal stem cells (MSCs) differentiated into neuron‐like cells via Notch signaling after lentivirus‐mediated upregulation of miR‐9.^6^ Nanoparticle‐based miRNA delivery systems have been tested for therapeutic applications.^7^ For example, Reid et al. delivered miR‐16 to mouse tumor tissues using bacteria‐derived nanoparticles, and demonstrated the delivered miRNA inhibited tumor growth.^8^ This gene delivery system is now being investigated in a Phase I clinical trial.^9^ Synthetic lipid nanoparticles have also been used for miRNA delivery to cells and tissues. The lipid bilayer of the nanoparticle can easily penetrate the plasma membrane into the recipient cell, and thus achieves efficient cellular uptake.^10^ Bader et al. delivered miR‐34a to lung tumors in mice using liposomes, and demonstrated 60% decrease in tumor area after delivery.^11^ The therapeutic effect of miR‐34a delivery is now being studied in a Phase I clinical trial using an amphoteric liposome.^12^ Although these results are promising, the majority of study to date has focused on cancer therapy. The number of reports on gene delivery to primary cells and stem cells is limited, due in part to the poor cellular uptake of miRNA.^13^, ^14^

Recently, synthetic cationic lipid‐like materials, called lipidoids, have been developed using a combinatorial library approach and used as delivery vehicles for a variety of payloads.^15^ Lipidoids can be synthesized easily and rapidly through a conjugate addition reaction. Lipidoids have been utilized for protein and small interfering RNA (siRNA) delivery applications in vivo with high efficiency.^15^, ^16^, ^17^, ^18^ More recently, our group has developed lipid nanoparticles containing disulfide bonds, which degrade in response to the intracellular environment. These lipid nanoparticle formulations promote the intracellular release of cargo and to reduce the off‐target immunostimulation and necrosis caused by the delivery.^19^ We have used these bioreducible lipids for delivery of siRNA and proteins.^19^, ^20^

MSCs have an ability to differentiate into many types of cells, including neuron‐like cells.^21^ MSCs are a valuable source for regenerative medicine, because autologous MSCs can be isolated relatively easily from the patient bone marrow or adipose tissues, as compared to neural stem cells (NSCs) which can be difficult to isolate.^22^ In addition, it takes relatively shorter time for MSCs to proliferate and differentiate into neuron‐like cells compared with induced pluripotent stem cells.^23^, ^24^, ^25^ In this study, we investigated the capability of the bioreducible lipids, synthesized using a combinatorial library, to deliver miRNAs to human mesenchymal stem cells (hMSCs). Notably, these cells are difficult to be transfected with nucleic acids. We used a fluorophore‐labeled miRNA and conducted screening assays of the lipids using microscopy and flow cytometry to identify an effective carrier in miRNA delivery. Then, we delivered miR‐9 to hMSCs and investigated its neuronal differentiation. This bioreducible lipid‐based miRNA delivery approach can be applied for nerve tissue engineering applications.

## 2 Results and discussion

### 2.1 Screening of bioreducible lipid‐based nanoparticles

**Figure 1:**
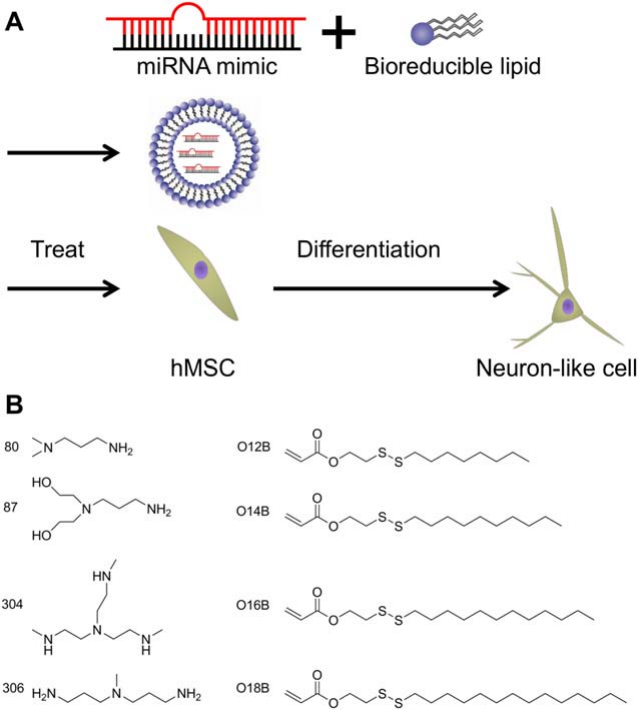
Schematic representation of this study. (A) Scheme of this study. Synthetic double‐stranded microRNA (miRNA mimic) was encapsulated in a bioreducible lipid by simply mixing in sodium acetate buffer. The nanoparticles were dosed to hMSCs and then neuronal differentiation was evaluated. (B) Chemical structures of amines and acrylates used for this study

Figure 1A illustrates the experimental design used to deliver miRNA to hMSCs and drive differentiation into neuron‐like cells using bioreducible lipid‐based nanoparticles. The bioreducible lipid and miRNA self‐assemble to form nanocomplexes through electrostatic interaction. The bioreducible lipids were synthesized according to our previous report.^19^ Briefly, amines and acrylates were mixed and heated at 80°C for 48 hr without any solvent. They were then cooled, and the crude products were used directly for the experiments. These lipids contain a disulfide bond which is degraded in the reductive intracellular environment, leading to the dissociation of the lipid–miRNA nanocomplex and release of the miRNA into the cytoplasm. This degradability also reduces the cytotoxicity of these lipids.^19^ In this study, we chose to screen bioreducible lipids made from a subset of our previously described library. Specifically, we tested combinations of four amines (80, 87, 304, and 306) and four acrylate tail lengths (O12B, O14B, O16B, and O18B) (Figure 1B). The four amines chosen have been previously demonstrated to effectively deliver siRNA to cancer cell lines.^18^, ^19^ Since miRNA and siRNA are similar in terms of structure, size, and charge to those of siRNA,^7^ we expected these bioreducible lipids to be effective in delivering miRNA as well.

**Figure 2:**
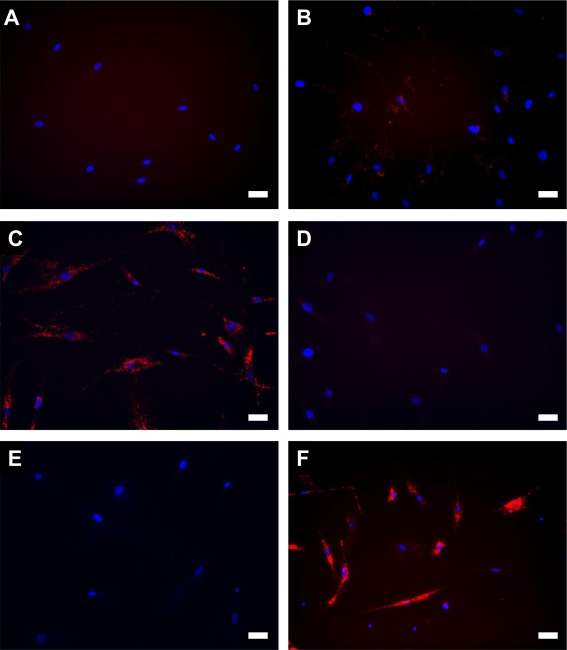
Delivery of Dy547‐labeled miRNA to hMSC. The cells were incubated for 4 hr after the treatment with red‐fluorescent dye (Dy547)‐labeled miRNA and then fixed with formalin. (A) miRNA alone, (B–F) miRNA encapsulated in (B) Lipofectamine 2000, (C) 80‐O16B, (D) 87‐O16B, (E) 304‐O18B, and (F) 306‐O16B‐3 bioreducible lipid. Blue, cell nuclei. Scale bar, 50 µm

First, we delivered a fluorescent dye (Dy547)‐conjugated miRNA for library screening and evaluated the delivery efficiency using a fluorescence microscope and a flow cytometer. Lipofectamine 2000 (LF2000), a commercial available lipid‐based nucleic acid delivery vehicle, was used as a control. To simplify our sample space, we chose to first identify the best amine head via a preliminary screening, and then to screen for tail length using only the ideal amine head. This approach has proven to be effective in our previous studies. First, we screened the efficacy of each of the four different amine heads in delivering miRNA. Cellular uptake of Dy547‐conjugated miRNA encapsulated in bioreducible lipids of each amine type was observed under a microscope (Figure 2). As expected, free miRNA was ineffective in entering the cells because of electrostatic repulsion between RNA molecules and cell membrane (Figure 2A). Under this experimental condition, LF2000 was not effective for delivery of the miRNA to hMSCs (Figure 2B). Fluorescence was observed best in cells treated with miRNA encapsulated in 306‐O16B‐3 lipid (Figure 2F). Considering this initial screening result, we proceeded to quantify delivery efficiency only with lipid containing 306 amine group.

**Figure 3:**
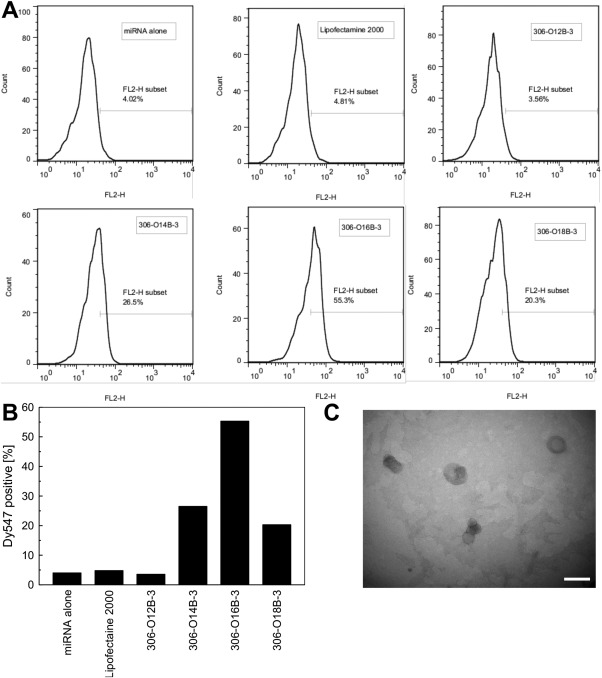
Bioreducible lipid delivers miRNA efficiently to hMSCs. (A, B) Quantification of Dy547‐labeled miRNA uptake by hMSCs using flow cytometry. The cells were cultured for 4 hr with the miRNA. Bioreducible lipids that have the same amine group (306) with different hydrophobic tail lengths (O12B‐3, O14B‐3, O16B‐3, and O18B‐3) were tested. As controls, Lipofectamine 2000 and free miRNA were used. (A) Typical output of flow cytometry. (B) Cellular uptake of miRNA was best when the miRNA was delivered using 306‐O16B‐3 bioreducible lipid. (C) TEM image of 306‐O16B‐3 bioreducible lipid–miRNA complex. Scale bar, 200 nm

Next, the uptake of Dy547‐labeled miRNA by hMSC was quantified using flow cytometry to determine the optimal tail length to deliver miRNA (Figures 3A and 3B). Bioreducible lipids synthesized by combining the 306 amine group with acrylates of different hydrophobic tail lengths (12, 14, 16, 18 carbons) were tested. As controls, LF2000 and free miRNA were used. The miRNA encapsulated in bioreducible lipids entered the cells efficiently, and the percentages of Dy547‐positive cells in bioreducible lipid groups were higher than those in control groups. Notably, the uptake efficiency changed with the length of the hydrophobic tail. Our result showed that lipids with a 16‐carbons tail showed higher delivery efficiency than lipids with 12, 14, or 18 carbon tail lengths. This result agrees with the report by Sun et al., who delivered DNA to HeLa cells using lipids.^26^ Sun et al. also showed that a 16‐carbon tail length achieved the best encapsulation efficiency. These results are possibly because long hydrophobic tails helped form stable nanoparticles with high solubility in cell membrane. On the other hand, Whitehead et al. reported tails with 12 or 13 carbons worked better than tail with 14 carbons for delivering siRNA to HeLa cells.^18^ In our study, the bioreducible lipid with a 12‐carbon tail (306‐O12B‐3) did not work for miRNA delivery, which agrees with our latest study for intracellular protein delivery.^20^ We have also recently demonstrated that the hydrophobic tail length of lipids affects the stability of nanoparticles in the presence of serum, and therefore the cellular uptake of nanoparticles.^20^ From these two screening experiments, we chose the chemical structure 306‐O16B‐3 for the use in the following experiments.

The 306‐O16B‐3–miRNA complex was further characterized by dynamic light scattering (DLS) and transmission electron microscopy (TEM). The size and zeta potential of 306‐O16B‐3 bioreducible lipid nanoparticles are shown in Table 1. The zeta potential decreased after the formation of lipid–miRNA complex, indicating that the complexation of miRNA neutralized the positive charge of the lipid. The nanoparticle morphology was visualized by TEM (Figure 3C).

**Table 1:** Physical properties of bioreducible lipid^a^

	Diameter [nm]	Zeta potential^b^ [mV]
306‐O16B‐3	213 ± 2.9	48.9 ± 8.4
306‐O16B‐3 + miRNA	533 ± 28.5	8.9 ± 1.6

a Size of bioreducible lipid nanoparticle was measured using dynamic light scattering.

b Zeta potential was also measured.

### 2.2 Neuronal differentiation of hMSCs induced by miR‐9 delivery

**Figure 4:**
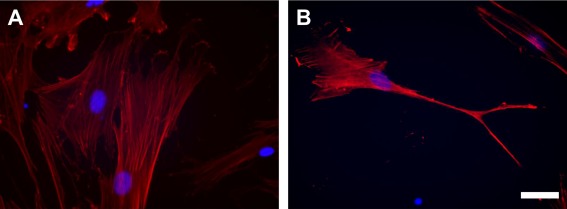
Cell morphology of hMSCs after 1 week of culture. Cells were stained using Fluor 555 Phalloidin (red). (A) Non‐treated hMSCs, (B) hMSCs treated with miR‐9 encapsulated in 306‐O16B‐3 bioreducible lipid nanoparticles. Blue: cell nuclei. Scale bar, 50 µm

Next, we treated hMSCs with miR‐9 encapsulated in 306‐O16B‐3 bioreducible lipid nanoparticles to differentiate hMSCs into neuron‐like cells. miR‐9 is known to promote neuronal differentiation of stem cells.^4^, ^6^, ^27^ Cells were cultured for 7 days following the delivery of miR‐9. No cytotoxic effect was observed during the culture. As shown in Figure 4, hMSC cell morphology changed in response to lipid nanoparticle‐mediated miR‐9 delivery. Lipid nanoparticle delivery of miR‐9 induced neuron‐like cell morphology.

**Figure 5:**
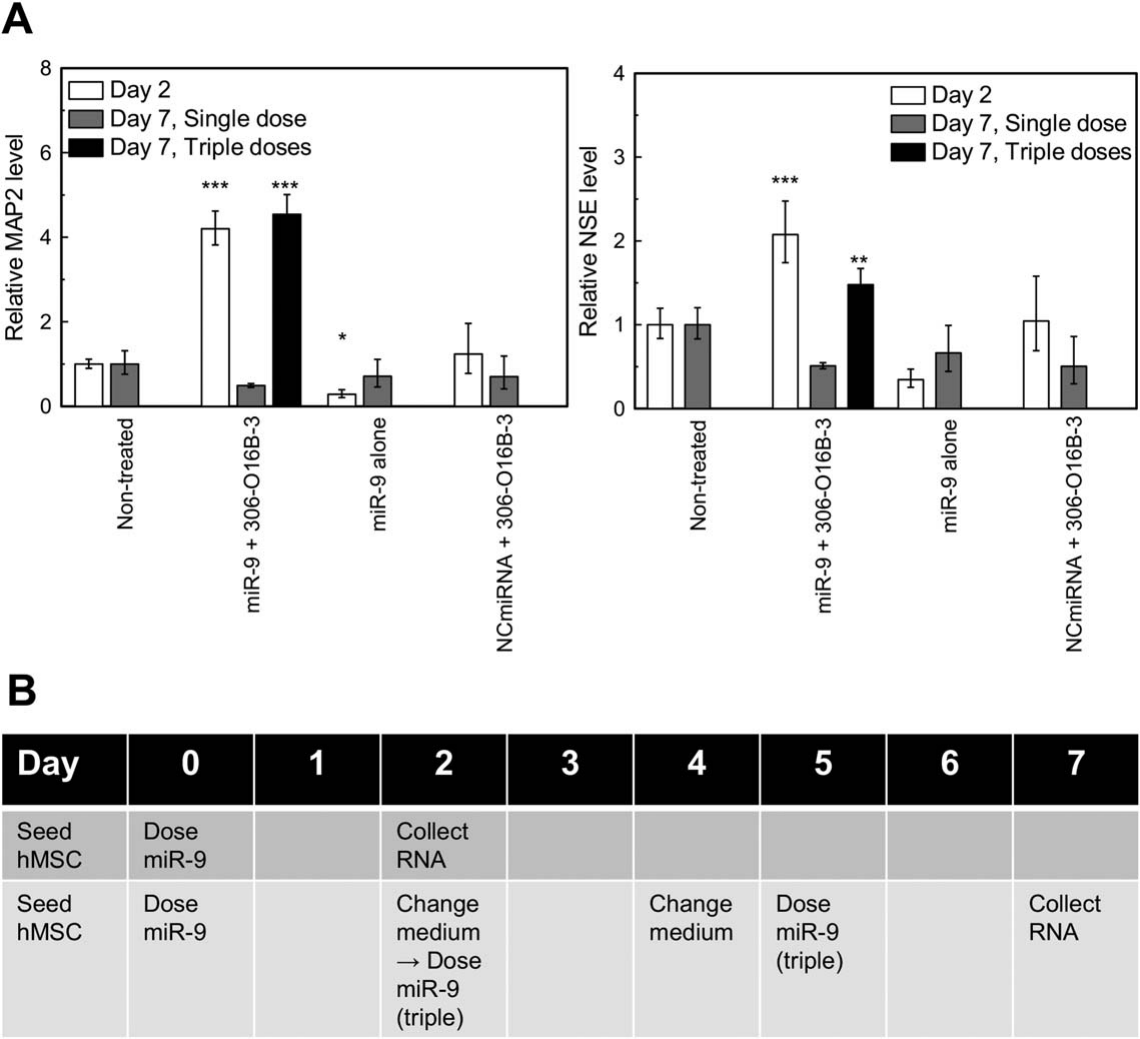
Quantitative PCR (qPCR) analysis. (A) mRNA expression of hMSC treated with miR‐9 encapusulated in bioreducible lipid nanoparticles for 1 week. Cells treated with free miR‐9 and with negative control miRNA (NCmiRNA) encapsulated in the lipid are shown as negative controls. **: *p* < .01, ***: *p* < .001, compared with non‐treated sample. All error bars represent standard deviation. MAP2: microtubule‐associated protein 2, NSE: neuron‐specific enolase. (B) Experimental timeline of multiple dosing of miR‐9 for qPCR. For the multiple dosage experiment, miR‐9 encapsulated in the bioreducible lipid was added to the culture medium on Day 0, 2, and 5

To confirm the effect of the lipid‐mediated miR‐9 delivery using lipid on neuronal differentiation, quantitative PCR (qPCR) was performed. Two days after delivery, mRNA expressions of microtubule‐associated protein 2 (MAP2) and neuron‐specific enolase (NSE) were upregulated (Figure 5A). In contrast, cells treated with free miR‐9 or with negative control miRNA (NCmiRNA) encapsulated in the lipid did not upregulate the neuronal marker genes. However, the upregulation of the genes was not observed 7 days after the delivery (Figure 5A; Day 7, Single dose). This result suggests that even though the cellular morphology changed with single lipofection, the effect of miRNA delivery on neuronal marker gene expressions was transient.

To maintain upregulation of the gene expression for 7 days, we increased the dosage frequency. Ma et al. demonstrated that increasing dosage frequency of lipid–siRNA complex improved knockout efficiency.^28^ In this study, following the first transfection on Day 0, miR‐9 encapsulated in the bioreducible lipid was also added to the culture medium on Days 2 and 5 (Figure 5B). As a result, the expressions of MAP2 and NSE remained upregulated on Day 7, and the expression levels were almost the same as those on Day 2 (Figure 5A; Day 7, Triple doses). This result suggests that multiple dosages of miRNA could maintain hMSCs differentiation. Further study is needed to determine how long the upregulated neuronal marker expressions are stable and to optimize the dose and dosage frequency.

## 3 Materials and methods

### 3.1 MSC culture

hMSCs were isolated from fresh bone marrow aspirate (Lonza) as previously described.^29^ hMSCs were expanded in Dulbecco's modified Eagle medium (DMEM) supplemented with fetal bovine serum (FBS) (10%), MEM Non‐Essential Amino Acids Solution (1%) (Gibco), penicillin streptomycin (PenStrep) (100 units/ml), and basic fibroblast growth factor (bFGF) (2 ng/ml) (Gibco). After expansion, hMSC was seeded on culture plates (Falcon, Tewksbury, MA) with a seeding density of 3.0 × 10^3^ cells/cm^2^. The hMSCs were cultured in DMEM supplemented with FBS (10%), MEM Non‐Essential Amino Acids Solution (1%), and PenStrep (100 units/ml). All hMSCs used for this study were passage 4.

### 3.2 Preparation of bioreducible lipids

Bioreducible lipids were synthesized using Michael addition conjugation as previously reported.^19^, ^26^ Amines and acrylates used in this study are shown in Figure 1B. Bioreducible lipids were named using the method of Wang et al.: the first number indicates the amine number, followed by an “O”, indicating acrylate, and the number of carbon atoms that comprises each hydrophobic tail of the acrylate. A “B” follows next, indicating a bioreducible lipid which contains disulfide bonds in the hydrophobic tail.^19^ Only lipids with amine 306 have three hydrophobic tails, and “−3” at the end of the name represents three hydrophobic tails. The other bioreducible lipids have two hydrophobic tails.

### 3.3 Screening of bioreducible lipid nanoparticles

Screening of bioreducible lipids was performed using fluorescent‐labeled miRNA. miRIDIAN microRNA Mimic Transfection Control with Dy547 (Dharmacon, Piscataway, NJ) was encapsulated in lipids by simply mixing in sodium acetate buffer (25 mm, pH = 5.2) and incubating at room temperature for 20 min. Encapsulated miRNA was then added directly to the culture medium of hMSCs in culture. The concentrations of miRNA and lipid were 20 pmol/ml culture medium and 1.5 µg/ml culture medium, respectively. As a control, Lipofectamine 2000 (Thermo Fisher Scientific, Grand Island, NY) was used according to the manufacturer's protocol. The cells were incubated for 4 hr at 37°C and then fixed with 10% formalin (Thermo Fisher Scientific). Cell nuclei were counterstained using Fluoroshield with DAPI (Sigma‐Aldrich). Cellular uptake of Dy547‐labeled miRNA was observed under a fluorescent microscope (Keyence, Itasca, IL). Uptake of miRNA by hMSCs was quantified with flow cytometry. After 4 hr of culture with the lipid–miRNA complexes, the cells were washed twice with phosphate buffered saline (PBS), then trypsinized, and reconstituted in PBS. The percentage of Dy547‐positive cells was counted using a BD FACSCalibur (BD Biosciences, San Jose, CA).

### 3.4 Characterization of nanoparticles

Diameter and zeta potential of bioreducible lipid were measured using a NanoBrook ZetaPALS (Brookhaven, Holtsville, NY). The lipid–miRNA complex was observed with TEM.

### 3.5 miRNA treatment with bioreducible lipid nanoparticles

hMSCs were seeded on a culture plate and allowed to attach overnight. On the next day (termed Day 0), synthetic hsa‐miR‐9 (Mission miRNA Mimic; Sigma‐Aldrich) was encapsulated in 306‐O16B‐3 bioreducible lipid and then delivered to the hMSCs. The concentrations of miRNA and lipid were 30 pmol/ml culture medium and 2.25 µg/ml culture medium, respectively. The cells were cultured for 1 week after the first delivery. The culture medium was changed every 2 days. For the multiple dosage experiment, miR‐9 encapsulated in the bioreducible lipid was added to the culture medium on Days 0, 2, and 5 (Figure 5C). As a negative control, ath‐miR‐416 (MISSION miRNA, Negative Control 1; Sigma‐Aldrich), which does not interact with any human genes, was delivered using the bioreducible lipid.

### 3.6 Cell morphological analysis

hMSCs in culture were treated with miRNA for 1 week, and then fixed with 10% formalin. The fixed cells were stained using Alexa Fluor 555 Phalloidin (Thermo Fisher Scientific). Cell nuclei were counterstained using Fluoroshield with DAPI. The images were taken with a fluorescent microscope.

### 3.7 Quantitative PCR (qPCR) analysis

qPCR was performed as described previously.^30^ Briefly, total RNA was isolated from hMSCs using miRNeasy micro kit (Qiagen, Limburg, Netherlands), and cDNA was synthesized from RNA by High‐Capacity cDNA Reverse Transcription Kit (Thermo Fisher Scientific). qPCR assay was performed using LightCycler 480 SYBR Green I Master (Roche, Basel, Switzerland). The mRNA expression level was normalized to that of GAPDH gene. Sequences of the primers (Integrated DNA Technologies, Coralville, IA) were as follows: MAP2 forward primer, GGAACCA ACTCTCTCTGGATTT; reverse primer, GCATTCTCTCTTCAGCCTTCT. NSE forward primer, CTGTATCGCCACATTGCTCAGC; reverse primer, AGCTTGTTGCCAGCATGAGAGC. GAPDH forward primer, ACCACA GTCCATGCCATCAC; reverse primer, TCCACCACCCTGTTGCTGTA. The differences were statistically evaluated using one‐way analysis of valiance (ANOVA) and Dunnett's post hoc test, which compared the values of miRNA‐treated samples with that of the non‐treated sample. Each experiment was performed in triplicate.

## 4 Conclusions

In this study, bioreducible lipid nanoparticles were developed for miRNA delivery to hMSCs. We demonstrated that bioreducible lipid nanoparticles can be used to deliver functional miRNAs to hMSCs. Optimal chemical structure for miRNA delivery to hMSCs was determined qualitatively and quantitatively. We also showed that miR‐9 encapsulated in the bioreducible lipid induced a change in the morphology of hMSCs and upregulated neuronal marker gene expression in the cells. It has been suggested that differentiated hMSCs could be applied for the treatment of nerve injuries and neurodegenerative diseases.^22^, ^31^ Our bioreducible lipid nanoparticles may provide an efficient way to generate neuronally differentiated hMSCs. In addition to neuronal differentiation, this bioreducible lipid nanoparticle has a potential to be applied to other therapeutic applications that utilize miRNA delivery to MSCs, such as stem cell‐based approaches to osteogenesis or seizure suppression.^14^, ^32^

## Disclosure statement

The authors have no conflict of interest.
